# The Proteomics and Metabolomics Analysis for Screening the Molecular Targets of Action of β-Eudesmol in Cholangiocarcinoma

**DOI:** 10.31557/APJCP.2021.22.3.909

**Published:** 2021-03

**Authors:** Kanawut Kotawong, Wanna Chaijaroenkul, Sittiruk Roytrakul, Narumon Phaonakrop, Kesara Na-Bangchang

**Affiliations:** 1 *Graduate Program in Bioclinical Sciences, Chulabhorn International College of Medicine, Thammasat University, Paholyothin Road, Klonglung, Pathumthani Thailand. *; 2 *Center of Excellence in Pharmacology and Molecular Biology of Malaria and Cholangiocarcinoma, Thammasat University, Paholyothin Road, Klonglung, Pathumthani Thailand. *; 3 *Center for Genetic Engineering and Biotechnology (BIOTEC), National Science and Technology Development Agency, Pathumthani, Thailand. *

**Keywords:** LC-MS/MS, Atractylodes lancea (Thunb) D.C., bile duct, cancer

## Abstract

**Objective:**

β-eudesmol is the active compound isolated from Atractylodes lancea (Thunb) D.C. The actions of this compound against cholangiocarcinoma (CCA) cells include anti-angiogenesis and anti-cell proliferation and growth. For more understanding of the molecular targets of action of β-eudesmol, the CCA cells (CL-6) were exposed to β-eudesmol for 24 and 48 hours.

**Methods:**

Proteins and metabolites from the intra- and extra-cellular components of the CL-6 cells were extracted and identified by LC-MS/MS. Protein analysis was performed using the Venn diagram (protein grouping), PANTHER (gene ontology), and STITCH software (protein-protein interaction). Metabolite analysis including their interactions with proteins, was performed using MetaboAnalyst software.

**Results:**

The analysis showed that the actions of β-eudesmol were associated with various biological processes particularly apoptosis and cell cycle. These included blood coagulation, wound healing, DNA repair, PI3K-Akt signaling pathway, immune system process, MAPK cascade, urea cycle, purine metabolism, ammonia recycling, and methionine metabolism.

**Conclusion:**

Possible molecular targets of action of β-eudesmol against CL-6 for cell apoptosis induction were TNFRSf6, cytochrome C, BAX3, DHCR24, CD29, and ATP. On the other hand, possible targets for cell cycle arrest induction were CDKN2B, MLF1, TFDP2, CDK11-p110, and nicotinamide.

## Introduction

**Figure 1:**
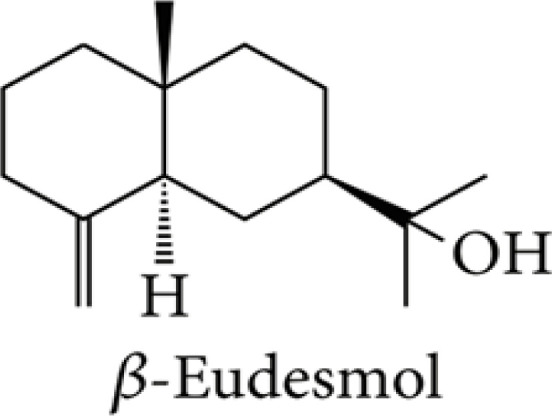
The Chemical Structure of β-eudesmol

Cholangiocarcinoma (CCA) is the cancer of the bile duct of which the highest incidence is reported in the northeastern region of Thailand. The main risk factor in the country is the consumption of improperly cooked fish containing Opisthorchis viverrini. Surgery, liver transplantation, chemotherapy, photodynamic therapy, and radiation therapy, are the key treatments for CCA, but their clinical efficacy and tolerability remain unsatisfactory. β-eudesmol (Figure 1) isolated from Atractylodes lancea (Thunb) D.C. (A. lancea) has been shown to be a promising candidate for further development for CCA chemotherapy (Na-Bangchang et al., 2017). The in vitro study demonstrated the mean (SD) IC50 (concentration that inhibits cell growth by 50%) of 39.33 (1.15) μg/ml in CCA cell line (Kotawong et al., 2018). Interruption of the cell cycle at G1 phase and cell apoptosis induction occurred after 24 and 48 hours of β-eudesmol exposure (Kotawong et al., 2018). A single and repeated oral dose (daily for 28-day) study in healthy mice showed the maximum tolerated and lethal doses of 100 and 200 mg/kg body weight, respectively (Plengsuriyakarn et al., 2015). The promising anti-CCA activity was demonstrated in CCA-xenografted mice, with a significant prolongation of survival time and survival rate compared with 5-fluorouracil-treated and untreated mice (Plengsuriyakarn et al., 2015). Apart from CCA, β-eudesmol has been reported to inhibit the proliferation of HeLa (cervical cancer), SGC-7901 (gastric cancer), and BEL-7402 (hepatoma) cells, as well as the Matrigel plugs angiogenesis in mice (Tsuneki et al., 2005; Ma et al., 2008). The compound has also been reported to suppress allergic reactions via inhibition of mast cell degranulation (Han et al., 2017) and migration (Nam et al., 2017), and to promote appetite through acting on the transient receptor potential ankyrin 1 (TRPA1) (Ohara et al., 2017). The present study aimed to explore molecular targets of action of β-eudesmol against CCA using proteomic and metabolomic approaches.

## Materials and Methods

*Cell culture*

CL-6, the human intrahepatic CCA cell line, was kindly provided by Associate Professor Dr. Adisak Wongkajornsilp, Department of Pharmacology, Faculty of Medicine (Siriraj Hospital), Mahidol University. The cell (106 /25 mm^2^ culture flask) was cultured in RPMI 1640 medium, supplemented with 10% (v/v) heated fetal bovine serum (FBS), and 100 IU/ml anti-biotic-antimycotic solution and maintained at 37^o^C, 5% CO_2 _atmosphere and 95% humidity for 24 hours. The cell was co-incubated with β-eudesmol at the concentration of 40 µg/ml (IC_50_) for 24 and 48 hours. After incubation, proteins and metabolites from intracellular (treated cell) and extracellular (culture media) were extracted and analyzed by LC-MS/MS.

*Proteomics*

Total protein concentrations from the intra- and extra-cellular components following exposure of CL-6 cells to β-eudesmol were measured using the Lowry method (Lowry et al., 1951). Proteins were separated using SDS-PAGE, digested with trypsin, and identified by LC-MS/MS. These identified proteins were grouped, and gene ontology and protein-protein interactions were analyzed using the Venn diagram, PANTHER and STITCH software, respectively.

*Protein Extractions*

For intracellular proteins, the CL-6 cells were washed with cold PBS and broken using SDS (0.5%) solution. Cell supernatant was collected through centrifugation at 800 xg (4°C) for 10 minutes and stored at -80ºC until use. For the extracellular proteins, the cell culture medium was mixed with pre-cold acetone (-20ºC) and incubated overnight at -20ºC. Proteins were precipitated through centrifugation (13,000 xg for 15 minutes), dissolved with SDS (0.5%), and stored at -80ºC until use. Protein concentrations in both intra- and extracellular components were measured using the Lowry method (Lowry et al., 1951).

*SDS-PAGE and In-Gel Digestions*

The extracted proteins from both intra- and extra-cellular components were separated based on the molecular weights using 12% SDS-PAGE at a constant current of 20 mA for 80 minutes. The gel containing proteins was stained by Coomassie Brilliant Blue R, cut into small fragments (19 fragments/sample), and transferred to a 96 well plate. Gel fragments were incubated with dithiothreitol (DTT) and iodoacetamide (IAA), digested with trypsin and extracted with acetonitrile. The extracted proteins from both the intra- and extracellular components of CL-6 cells were dried at 40°C (overnight) and stored at -80°C until use (Kotawong et al., 2015).

*LC-MS/MS Analysis*

The dried-proteins from each gel fragment were dissolved with 0.1% formic acid and injected onto LC-MS/MS for protein identification. The chromatographic conditions consisted of a μ-pre-column (Monolithic Trap Column, 200 μm i.d. × 5 mm), a nano column (Monolithic Nano Column, 100 μm i.d.× 5 cm) coupled with the Ultimate 3000 LC system (Dionex), and ESI-Ion Trap MS (HCT ultra PTM Discovery System, BrukerDaltonik) with electrospray at a flow rate of 20 μl/minute. The solvent gradient mobile phase consisted of solvent A (0.1% formic acid) and solvent B (50% water, 50% acetonitrile, 0.1% formic acid) running at a flow rate of 1 µl/min with 20 minutes run time.

*Protein Identification and Analysis*

The LC-MS/MS data were analyzed using DeCyderTM (Amersham Bioscience AB, Uppsala, Sweden) and MASCOT (http://www.matrixscience.com) programs for protein identification. PANTHER (http://www.pantherdb.org) and STITCH software (http://stitch.embl.de) were respectively applied for the identification of gene ontology and protein-protein interactions

*Metabolomics*

The CL-6 cells were incubated with β-eudesmol (40 µg/ml) for 24 and 48 hours. The metabolites from both CL-6 (intracellular) and culture medium (extracellular) were extracted and identified by XcalibarTM software. After metabolite identification, MetaboAnalyst 4.0 software was applied for the analysis of the metabolic network.

*Metabolite Extraction*

To identify intracellular metabolites, the β-eudesmol-exposed CL-6 cell was washed with cold PBS and incubated with 80% methanol at -80°C for 20 minutes. The cell was then transferred to a new 15 ml-test tubes using cell scraper and centrifuged at 14,000 xg (4-8°C) for 5 minutes. The supernatant was collected, dried (SpeedVac), and stored at - 80°C until use (Yuan et al., 2012). For the extracellular metabolites, the culture media was collected after cell exposure and mixed with methanol (cooled to -80°C) and incubated at -80°C for 8 hours. Cell supernatant was collected through centrifugation at 14,000 xg (4-8°C) for 10 minutes, dried (SpeedVac) and stored at -80°C until use (Yuan et al., 2012).

*Metabolite Identification*

The dried metabolites from both intra- and extra-cellular were dissolved with LC/MS grade water (500 µl) and filtered through Phenix-RC filter (0.2 µm pore size). The metabolites were isolated using HPLC (Agilent Technologies, 1200 Infinity Series) and identified by QTRAP^®^ 5500 (AB SCIEX) (Yuan et al., 2012). The system consisted of security universal HPLC guard cartridge (Phenomenex, CA, USA), amide XBridge HPLC column (3.5 μm x 4.6 mm i.d. × 100 mm length (Waters, MA, USA). The mobile phase consisted of 50% buffer A (95% water, 5% acetonitrile, 20 mM ammonium hydroxide, and 20 mM ammonium acetate adjusted to pH 9.0) and 50% of buffer B (100% acetonitrile). The flow rate was 400 µl/minute with a run time of 23 minutes. For MS/MS, the spray voltage was set at 3,200 V. Nitrogen and argon were used as auxiliary and collision gas, respectively. Scan time for each single-reaction monitoring (SRM) event transition was 0.1 second with a scan width of 1 m/z.

*Metabolomics Analysis*

The instrument control, data acquisition, and data analysis were achieved using XcalibarTM software (Thermo Scientific, Massachusetts, USA). The metabolites which were up- and down-regulated were selected for identification of possible signaling pathways including the correlation between the identified metabolites and proteins using the free online web-based MetaboAnalyst 4.0 (http://old.metaboanalyst.ca/MetaboAnalyst/ faces/ ModuleView. xhtml)

## Results

*Proteomics*

**Figure 2.**
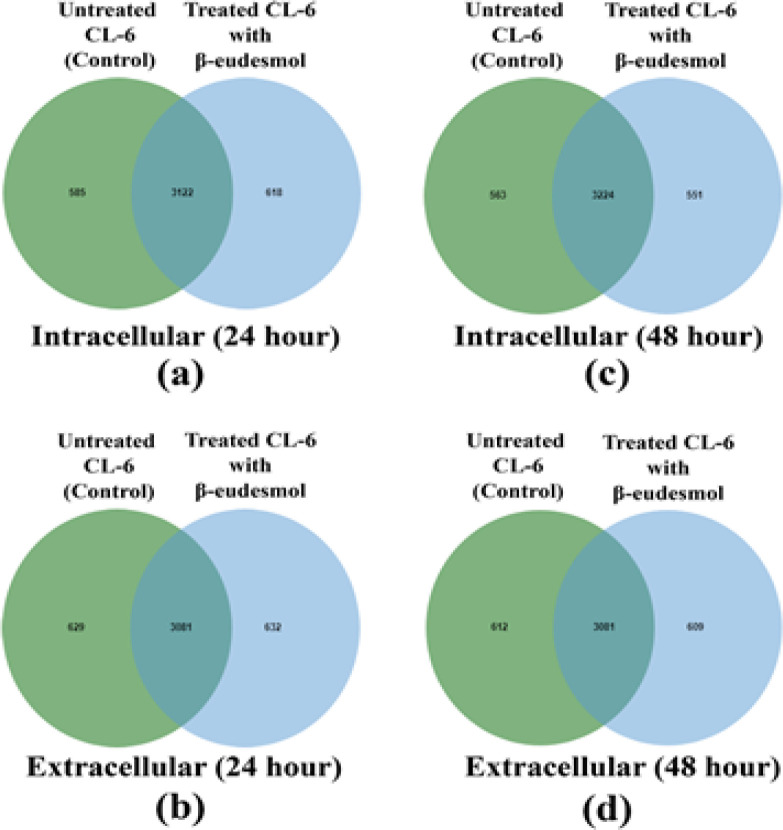
Venn Diagrams of Intracellular Proteins at 24 hour (a), extracellular proteins at 24 hours (b), intracellular proteins at 48 hours (c), and extracellular proteins at 48 hours (d) following exposure of CL-6 cells to β-eudesmol

**Figure 3:**
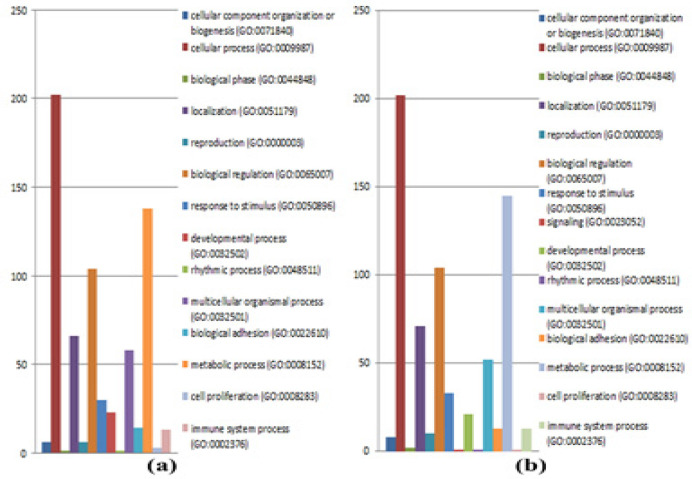
Protein Classification Based on the Biological Processes of Proteins Identified at 24 hours (a) and 48 hours (b).

**Figure 4:**
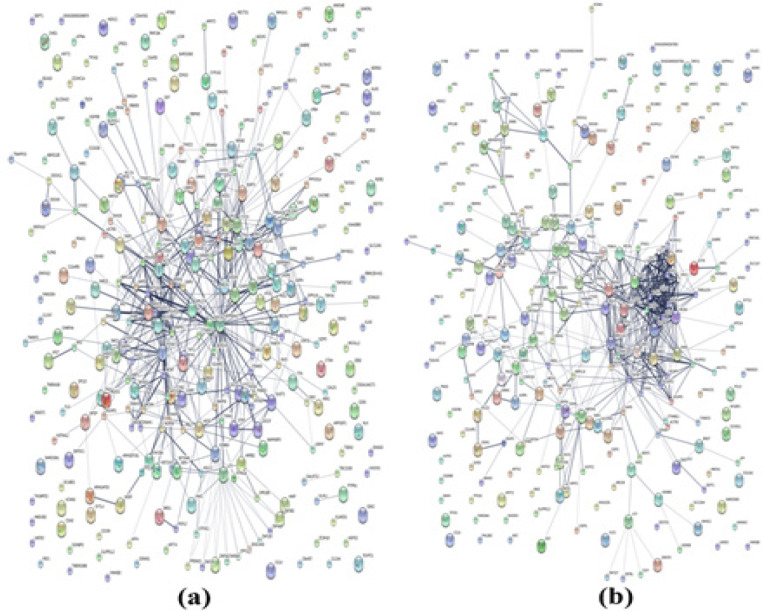
The Protein-Protein Interactions Analysis by SITTCH of Proteins Identified at 24 hours (a) and 48 hours (b) following exposure of CL-6 cells to β-eudesmol

A total of 4,325 and 4,342 proteins were identified from the intra- and extracellular components of the CL-6 cells following exposure to β-eudesmol for 24 hours. These proteins were divided into three groups based on the analysis of Venn diagram as proteins that were found only in untreated cells (585 and 629 proteins for the intra- and extracellular components, respectively), or only in β-eudesmol-treated cells (618 and 632 proteins for the intra- and extracellular components, respectively), or both in the untreated and treated cells (3,122 and 3,081 proteins for intra- and extracellular components, respectively) (Figure 2a and 2b). The proteins that were found only in the untreated and treated cells were further selected for gene ontology analysis by PANTHER software. The analysis identified 671 proteins involved in biological processes. Most (202 proteins) are involved in cellular processes (GO:0009987), followed by the metabolic process (GO:0008152, 138 proteins), and biological regulation (GO:0065007, 104 proteins) (Figure 3a). Besides, 26 proteins are transporters, i.e., amiloride-sensitive sodium channel subunit gamma, calcium-activated chloride channel regulator 4, calcium-binding mitochondrial carrier protein SCaMC-3, caprin-2, and gamma-aminobutyric acid receptor subunit epsilon (SSD 1). Analysis of protein-protein interactions by STITCH software identified their roles in the cell cycle, apoptosis, PI3K-Akt signaling pathway, DNA replication, blood coagulation, DNA repair, wound healing, and cell differentiation (Figure 4a).

For the 48 hour exposure, 4,338 and 4,302 proteins were identified from the intra- and extracellular components of the CL-6 cells. Based on the Venn diagram analysis, 563 and 612 proteins, respectively, were found only in the intra- and extracellular components of the untreated cells. Five hundred and fifty-one and 609 proteins, respectively, were found only in the intra- and extracellular components of the β-eudesmol-treated cells (Figures 2c and 2d). The corresponding numbers of proteins found in the intra- and extracellular components of both the untreated and treated cells were 3,224 and 3,018, respectively. Further analysis of proteins that were found only in the untreated and treated cells of both intra- and extracellular components by PANTHER software suggested their involvements in cellular process (GO:0009987, 202 proteins), metabolic process (GO:0008152, 145 proteins), biological regulation (GO:0065007, 104 proteins), and localization (GO:0051179, 71 proteins) (Figure 3b). Besides, 18 proteins are identified as transporters, i.e., ATP-binding cassette sub-family G member 8, calcium-binding mitochondrial carrier protein SCaMC-3, cystic fibrosis transmembrane conductance regulator, excitatory amino acid transporter 5, and gamma-aminobutyric acid receptor subunit epsilon (SSD 1). Analysis of protein-protein interactions by STITCH software identified their roles in DNA replication, cell cycle, cellular response to DNA damage stimulus, DNA repair, and response to stress (Figure 4b).

*Metabolomics*

**Figure 5:**
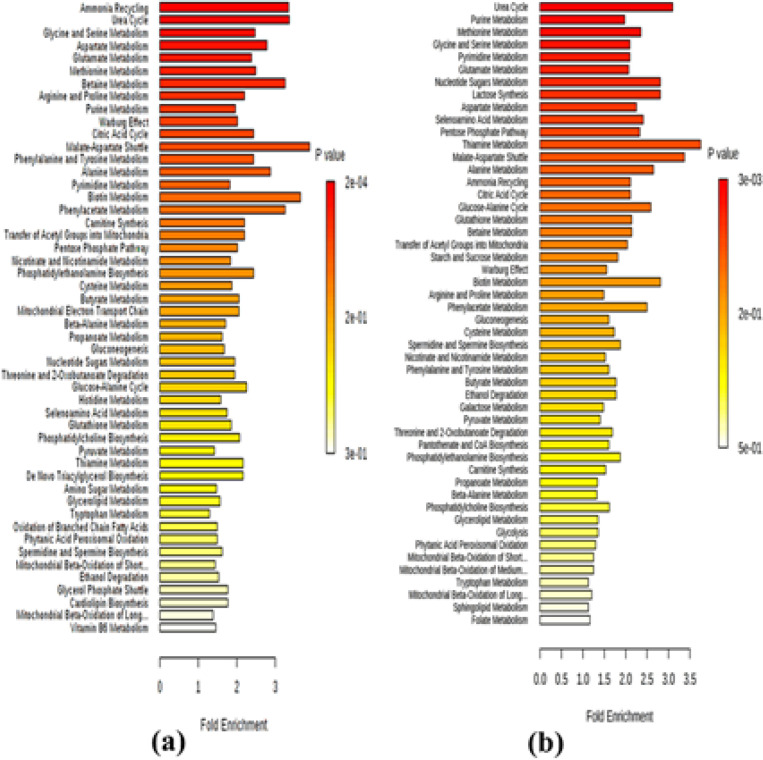
Analysis (by MetaboAnalyst Software) of Metabolites Identified Following Exposure of CL-6 Cells to β-Eudesmol for 24 hours (a) and 48 (b) hours

The area under the peak of each metabolite identified by LC-MS/MS was calculated and compared with control (untreated cell). Metabolites with the change in the peak area of at least 2-fold were selected for further analysis. For 24 hour incubation, the peak areas of 9, 86, 38 and 38 metabolites had at least 2-fold change, i.e., up- and down-regulated intracellular, and up- and down-regulated extracellular metabolites, respectively (SSD 2). The highest change was found with glutathione disulfide (90.75 fold), followed by cytidine (28.29 fold), deoxyribose-phosphate (26.64 fold), and choline (19.65 fold). The top five signaling pathways involved with these metabolites identified by MetaboAnalyst software were ammonia recycling (11 metabolites), urea cycle (10 metabolites), glycine and serine metabolism (15 metabolites), aspartate metabolism (10 metabolites), and glutamate metabolism (12 metabolites) (Figure 5a). For 48 hour incubation, the peak areas of 71, 10, 16 and 43 metabolites had at least 2-fold change, i.e., up- and down-regulated intracellular, and up- and down-regulated extracellular metabolites, respectively (SSD 3). The highest change was found with glutathione disulfide (143.95 fold), followed by GMP (63.20 fold), taurodeoxycholic acid (35.69 fold), acetyl phosphate (25.89 fold), and shikimate-3-phosphate (21.38 fold). The signaling pathways involved with these metabolites were urea cycle (8 metabolites), purine metabolism (13 metabolites), methionine metabolism (9 metabolites), glycine and serine metabolism (11 metabolites), and pyrimidine metabolism (11 metabolites) (Figure 5b).

*Co-Analysis of Proteins and Metabolites*

**Figure 6:**
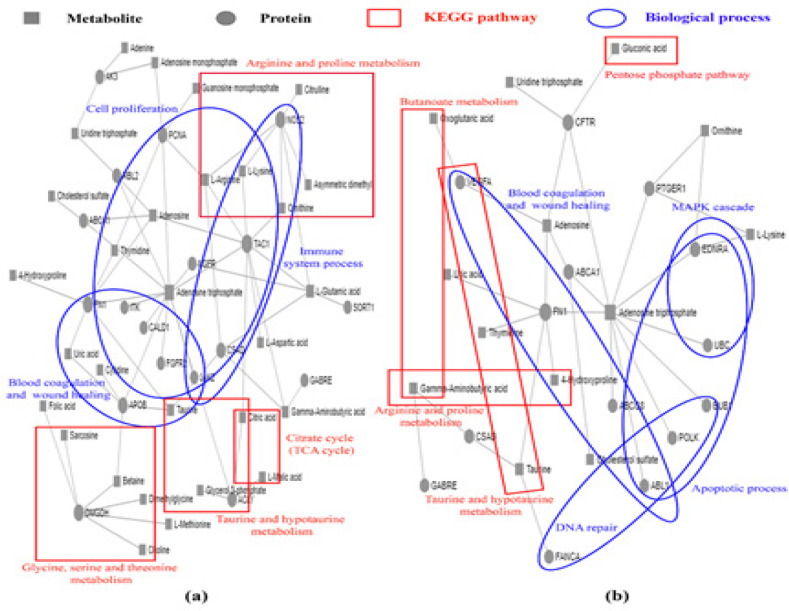
Co-Analysis of Protein and Metabolite Interactions (by Metabo Analyst Software) Following Exposure of CL-6 Cells to β-eudesmol for 24 hours (a) and 48 hours (b).

The proteins detected from both intra- and extracellular components only in the untreated and β-eudesmol-treated CL-6 cells at 24 and 48 hours, and the metabolites with at least 2-fold changes in peak areas were used for the co-analysis of the interactions associated signaling pathways using MetaboAnalyst software. For 24 hour incubation, 18 proteins and 29 metabolites were involved in the interactions (Figure 6a). The KEGG pathway of this interaction was involved with arginine and proline metabolism, glycine, serine and threonine metabolism, aminoacyl-tRNA biosynthesis, taurine, and hypotaurine metabolism, and citrate cycle (TCA cycle). Besides, the biological process involved in these interactions was associated with blood coagulation, wound healing, immune system process, and cell proliferation. For 48 hour incubation, 14 proteins and 13 metabolites were involved in the interaction (Figure 6b). These signaling pathways were involved with taurine and hypotaurine metabolism, arginine and proline metabolism, butanoate metabolism, alanine, aspartate and glutamate metabolism, and biotin metabolism. Moreover, regulation of MAPK cascade, blood coagulation, wound healing, apoptotic process, and DNA repair were also involved in these interactions.

**Figure 7:**
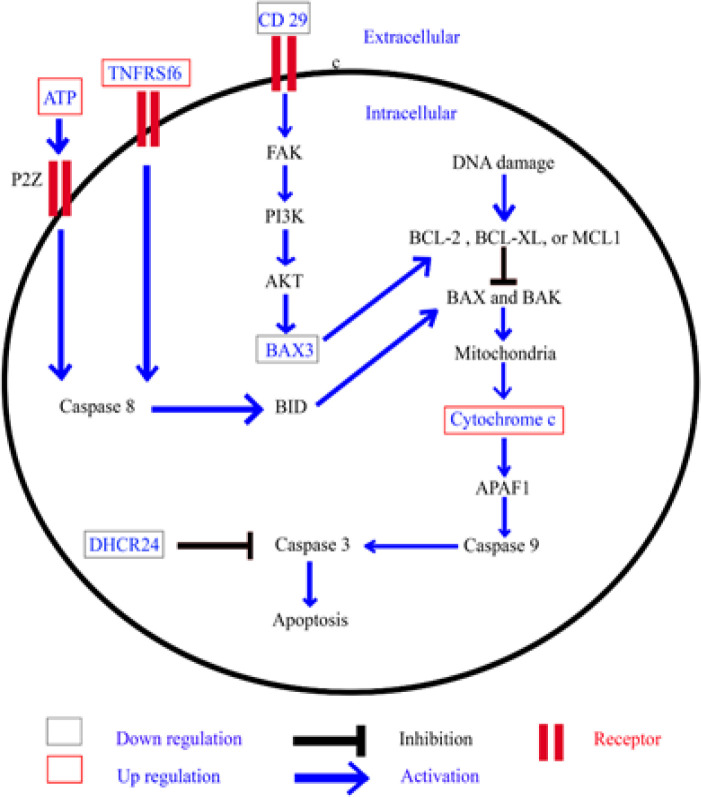
Proposed Molecular Targets of Anti-CCA Actions of β-eudesmol through the Induction of Cell Apoptosis

**Figure 8:**
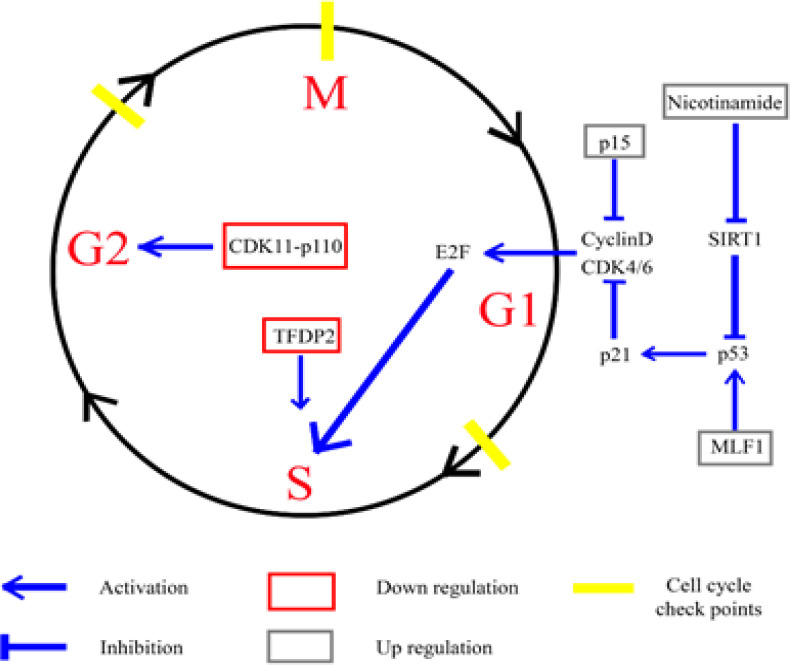
Proposed Molecular Targets of Anti-CCA Actions of β-eudesmol through the Induction of Cell Cycle Arrest

## Discussion

Major signaling pathways associated with the molecular signaling targets of anticancer drugs include cell proliferation, cell apoptosis, metastasis, angiogenesis, and immunomodulation. The present study focused on apoptosis and cell proliferation as molecular signaling pathways of the anti-CCA action of β-eudesmol (Kotawong et al., 2018).

For apoptosis regulation, the proteins that were found only in control or only in β-eudesmol-treated CL-6 cells were selected to identify the molecular targets of action of β-eudesmol associated with the apoptosis process. Apoptosis, the programmed cell death found in both normal and abnormal cells involves two processes, i.e., intrinsic and extrinsic pathways (Elmore, 2007). The intrinsic pathway is induced by DNA damage from heat, radiation, and chemical exposure including chemotherapeutic drugs. Various signaling pathways are activated through the p53, followed by bcl2, bax, cytochrome c (Cytc), caspase 9, caspase 3, and apoptosis (Loreto et al., 2014). The extrinsic pathway, on the other hand, is induced by death cell receptors, e.g., Fas receptor (FasR), TNF-related apoptosis-inducing ligand (TRAIL) receptors, and tumor necrosis factor receptor (TNFR) (Fulda and Debatin, 2006). Following receptor activation, caspase 8 and caspase 3 signalings are further activated. The intrinsic and extrinsic pathways are linked together via bid protein. Results of the present study showed that the molecular target proteins of β-eudesmol associated with cell apoptosis were TNF superfamily, member 6 (TNFRSf6) (up-regulation), Cytc (up-regulation), Bcl2-associated athanogene 3 (BAX3) (down-regulation), 24-dehydrocholesterol reductase (DHCR24) (down-regulation), and β1-integrin (CD29) (down-regulation). The TNFRSf6 (CD95, or Apo-1) is a death cell receptor that is located on the cell surface. High expression of TNFRSf6 was observed after the exposure of ataxia telangiectasia and human cervical cancer cells (Albanese and Dainiak, 2000) to radiation and pinostrobin (bioflavonoid in dietary and herb) (Jaudan et al., 2018), respectively. Receptor activation leads to cell apoptosis via the extrinsic pathway (caspase 8 activation). Cytc, the hemoprotein located in cell mitochondria, plays role in mitochondrial electron transport, cardiolipin peroxidation (Kagan et al., 2005), ROS formation, and cell apoptosis (Cai et al., 1998). Regarding cell apoptosis, several compounds such as tamoxifen (an anticancer drug for breast cancer) and botulin (triterpene from plants, e.g., birch trees) induce the release of Cytc from mitochondria in a dose-dependent manner (Hassan et al., 2018; Zhou et al., 2018). BAG3, the protein belonged to the member of BAG family, plays a role in the protection of cells from the apoptosis process by stabilizing anti-apoptotic Bcl-2 family proteins as bcl-2 and bcl-XL (Kim et al., 2013). The decrease in BAG3 expression leads to cancer cell apoptosis. High expression of BAG3 was shown to be associated with the induction of cell proliferation, migration, and invasion in colorectal cancer (Li et al., 2018), but with the suppression of apoptosis process in MCF7 cells (Pasillas et al., 2015). DHCR24, a member of the flavin adenine dinucleotide (FAD)-dependent oxidoreductases, plays a critical role in regulating oxidative stress, inflammatory, and cell apoptosis. Increased expression of DHCR24 suppressed apoptosis process by reducing caspase 3 activation (Greeve et al., 2000; Lu et al., 2012). Abnormality of this protein has been associated with cardiovascular diseases (Wu et al., 2013) and hepatitis C (Takano et al., 2011). The cell surface receptor CD29 regulates tumor growth (activation), metastasis (activation), and apoptosis (suppression) (Howe and Addison, 2012; Zhang et al., 2015). The suppression of cell apoptosis as a result of β1-integrin activation is through FAK/PI3K/AKT pathway (Nguyen et al., 2016). The metabolite that was shown to be involved with CL-6 apoptosis was adenosine triphosphate (ATP), a molecule that carries energy within cells. The increase in ATP expression in extracellular component induces apoptosis via P2Z receptor, followed by caspase 8, and caspase 3 (Ferrari et al., 1999; Wen and Knowles, 2003).

Altogether, results suggested that β-eudesmol induced CL-6 cell apoptosis through both intrinsic and extrinsic pathways. Possible molecular targets in the intrinsic pathway were Cytc and BAX3, while those of the extrinsic pathway were TNFRSf6, β1-integrin and adenosine triphosphate (Figure 7).

For cell proliferation regulation, cell proliferation is the cellular processes involved in cell division. The process is regulated by three cell cycle checkpoints (Barnum and O’Connell, 2014). The first checkpoint occurs at the G1 phase which involves cyclin D, cyclin E, CDK2, CDK4, p53, RB, and E2F as the main proteins (Bertoli et al., 2013). Abnormality of these proteins results in cell cycle arrest at the G1 phase. The second checkpoint occurs at the G2/M phase which involves cyclin B and CDK1 as major proteins (DiPaola, 2002). This checkpoint functions to verify the chromosome number and necessary materials before cell division. The third checkpoint occurs at the cell division M phase to control chromosome alignment and segregation (Barnum and O’Connell, 2014). In the present study, the proteins involved in cell cycle which were found after exposing the CL-6 cells to β-eudesmol were cyclin-dependent kinase inhibitor 2B (CDKN2B) (up-regulation), myeloid leukemia factor 1 (MLF1) (up-regulation), transcription factor Dp-2 (TFDP2) (down-regulation), and cyclin-dependent kinase 11A (CDK11-p110) (down-regulation), while the involved metabolite was nicotinamide (up-regulation). The tumor suppressor protein CDKN2B (p15) induces cell cycle arrest at G1 phase by inhibition of cyclin D complex activity. Besides, the increase in CDKN2B expression has been shown to increase the sensitivity of hepatocellular carcinoma to sorafenib (Weng et al., 2019). MLF1 is located in the nucleoplasm and regulates both cell cycle and apoptosis processes. An increase in the expression of this protein has been shown to induce cell cycle arrest at G1 phase through p53 and p21 activation (Yoneda-Kato et al., 2005) while inducing cell apoptosis via an intrinsic pathway through suppression of bcl-XL (Sun et al., 2015). TFDP2 is the important protein involved in cell division and is activated by the transcription factor E2F2. The decrease in the expression of this protein has been shown to result in the activation of cell cycle arrest at S phase during erythropoiesis (Chen and Lodish, 2014). The CDK11-p110 (known as CDC2L2, CDC2L3, p58GTA, PITSLRE, CDK11-p46, or CDK11-p58) regulates cell proliferation. Downregulation of this protein has been shown to induce cell cycle arrest at G1 phase in human breast cancer cells (Zhou et al., 2015), and at G2/M phase in esophageal squamous cell carcinoma (Du et al., 2019). Moreover, the induction of apoptosis in esophageal squamous cell carcinoma was observed when CDK11-p110 was inhibited by RNA interference (Du et al., 2019). In the present study, the active form of vitamin B3, nicotinamide (known as niacinamide), was shown to be the metabolite that is linked with the cell cycle. The increasing of nicotinamide activated cell cycle arrest via SIRT1 suppression following activation of p53 and p21, respectively (Dell’Omo and Ciana, 2019). The apoptosis of intrahepatic CCA was shown to be induced by this metabolite (Wang et al., 2018).

Altogether, results suggested that the action of β-eudesmol on CL-6 cell proliferation was through cell cycle arrest at G1, S, and G2/M phase. The signaling at the G1 phase was through the activation of MLF1, followed by p53 and p21, respectively. Activation of p21 resulted in suppression of the activity of cyclin D and E complex and, thus, cell cycle arresting at G1 phase. Besides, the activity of cyclin D and E complexes was also inhibited through the activation of CDKN2B (p15) and nicotinamide. For the cell cycle arrest at S and G2/M phases, activation of the signaling pathways of the protein targets (TFDP2 and CDK11-p110) by β-eudesmol remains unclear (Figure 8).

In conclusion, the application of proteomic and metabolomic approaches in this study revealed key signaling pathways associated with molecular targets of anti-CCA action of β-eudesmol, particularly those involved in apoptosis and cell cycle. These signaling pathways were associated with blood coagulation, wound healing, DNA repair, PI3K-Akt signaling, immune system process, MAPK cascade, urea cycle, purine metabolism, ammonia recycling, and methionine metabolism. For apoptosis regulation, possible molecular targets included TNFRSf6, Cytc, BAX3, DHCR24, CD29, and ATP. Possible molecular targets for cell cycle arrest induction included CDKN2B, MLF1, TFDP2, and CDK11-p110, and nicotinamide.

## Author Contribution Statement

Kanawut Lotawong was involved in conducting laboratory experiments, data analysis, and drafting the manuscript. Wanna Chaijaroenkul was involved in study supervision, and data analysis. Sittiruk Roytrakul was involved in conceptualization of the study, and study supervision. Narumon Phaonakrop was involved in conducting laboratory experiments, and data analysis. Kesara Na-Bangchang was involved in conceptualization of the study, study supervision, and revising the manuscript. All finalized and approved the last version of the manuscript.

## Study Approval

The study is part of an approved student’s dissertation (Mr. Kanawut Kotawong) by the Graduate Studies, Chulabhorn International College of Medicine, Thammasat University.
